# Study of the regulatory elements of the *Ovalbumin* gene promoter using CRISPR technology in chicken cells

**DOI:** 10.1186/s13036-023-00367-3

**Published:** 2023-07-17

**Authors:** Sara Yousefi Taemeh, Nima Dehdilani, Lena Goshayeshi, Sylvie Rival-Gervier, Jalil Mehrzad, Bertrand Pain, Hesam Dehghani

**Affiliations:** 1 Division of Biotechnology, Faculty of Veterinary Medicine, Ferdowsi University of Mashhad, Mashhad, Iran; 2 Stem Cell Biology and Regenerative Medicine Research Group, Research Institute of Biotechnology, Ferdowsi University of Mashhad, Mashhad, Iran; 3 Stem Cell and Brain Research Institute, University of Lyon, Université Lyon 1, INSERM, INRAE, U1208, USC1361, Bron, 69500 France; 4 Department of Microbiology and Immunology, Faculty of Veterinary Medicine, University of Tehran, Tehran, Iran; 5 Department of Basic Sciences, Faculty of Veterinary Medicine, Ferdowsi University of Mashhad, Mashhad, Iran

**Keywords:** Chicken fibroblast, *Ovalbumin* promoter, CRISPR technology, Avian expression systems, Regulatory sequences, Gene editing

## Abstract

**Background:**

Hormone-dependent promoters are very efficient in transgene expression. Plasmid-based reporter assays have identified regulatory sequences of the *Ovalbumin* promoter that are involved in response to estrogen and have shown that the deletion of the steroid-dependent regulatory element (SDRE) and negative regulatory element (NRE) leads to a steroid-independent expression of a reporter. However, the functional roles of these regulatory elements within the native genomic context of the *Ovalbumin* promoter have not been evaluated.

**Results:**

In this study, we show that the negative effects of the NRE element on the *Ovalbumin* gene can be counteracted by CRISPR interference. We also show that the CRISPR-mediated deletion of SDRE and NRE promoter elements in a non-oviduct cell can lead to the significant expression of the *Ovalbumin* gene. In addition, the targeted knock-in of a transgene reporter in the *Ovalbumin* coding region and its expression confirms that the truncated promoter of the *Ovalbumin* gene can be efficiently used for an estrogen-independent expression of a foreign gene.

**Conclusions:**

The methodology applied in this paper allowed the study of promoter regulatory sequences in their native nuclear organization.

**Supplementary Information:**

The online version contains supplementary material available at 10.1186/s13036-023-00367-3.

## Background

Avian expression systems represent desirable platforms for the production of recombinant human proteins. Production in chicken cells offers significant advantages over other systems, including providing human-like glycosylation on target proteins [1]. In the early attempts to produce foreign proteins in avian systems, viral vectors containing a constitutive promoter, such as a cytomegalovirus (CMV) promoter, were utilized to drive expression [2, 3]. However, the utilization of these constitutive/strong promoters had several disadvantages including variations in protein expression levels, improper folding of the protein product, promoter silencing possibilities, and toxicity arising from their expression in a broad range of tissues [4–6]. Thus, there has been an increasing trend toward the use of regulated promoters. Among these, native hormone-dependent promoters have been demonstrated to be efficient in transgene expression. One such example is the *Ovalbumin* (*OVA*) promoter, which has been used in cultured primary oviduct cells or transgenic chickens for the production of exogenous proteins [7–17].

Seven members of the chicken clade B serpins have orthologues in the human genome. However, the *Ovalbumin* gene (*SerpinB14*) and two of its paralogues, the *Ovalbumin-related protein Y* (*SERPINB14B*) and the *Ovalbumin-related protein X* (*SERPINB14C*) do not have any orthologues in the human genome [18]. The expression of these three genes, in contrast to other serpins, is hormone-dependent [19]. Consistent with this, the regulatory elements of these genes do not exhibit significant similarity to the regulatory regions of other serpins. The sequences of the steroid-dependent regulatory element (SDRE; − 900 to − 732) and negative regulatory element (NRE; − 308 to − 88) are unique to the chicken *OVA* gene. Several plasmid-based reporter assays have been used to elucidate the role of the regulatory elements within the *OVA* promoter [20–31]. These studies have demonstrated that the presence of a proximal promoter (− 87 to + 9) is sufficient for steroid-independent expression. The deletion of the SDRE and NRE, along with the linker between them, in the chicken *OVA* promoter resulted in increased activity of the reporter gene [24, 27, 28]. However, it is important to identify additional distant regulatory elements that are associated with the oviduct-specific function of the *OVA* promoter [10].

To induce the expression of exogenous genes in plasmid constructs, researchers have utilized different 5' and 3' flanking regions of the chicken *OVA* promoter. Some studies suggest that incorporating the two key regulatory elements, SDRE, and NRE, present within the chicken *OVA* promoter is sufficient to achieve oviduct-specific expression of a therapeutic protein [8, 16]. These two regulatory elements are critical for the appropriate regulation of the *OVA* gene expression [24, 25, 32–34]. The SDRE is essential for the response to steroid hormones, including estrogen, progesterone, androgen, and glucocorticoids [21]. On the other hand, the NRE acts as a bifunctional element. It collaborates with SDRE to activate *OVA* gene expression in the presence of steroids in the oviduct tissue while repressing *OVA* gene transcription in the absence of steroids in both oviduct and non-oviduct cells [24, 27–29]. It has been demonstrated that a specific element within the NRE, known as the COUP adjacent repressor (CAR) element (-130 to -100), plays a major role in mediating the repressive activities of the NRE [28, 29]. Another negative element within the NRE is a ubiquitous silencer (-239 to -220), which leads to a reduction in transcriptional activity by approximately three-fold and acts as a genuine transcriptional silencer since it is capable of repressing a heterologous promoter in an orientation-independent manner [27].

In an attempt to improve the expression level of the transgene in a non-native genomic site or a plasmid construct, additional regulatory sequences comprising the *OVA* exon 1, intron 1, and the beginning of exon 2 were incorporated into the promoter construct [8]. Zhu et al. utilized 7.5 kb and 15 kb of the 5' flanking region, as well as 15.5 kb of the 3' flanking region from the *OVA* gene to direct *ex-situ* transgene expression [7]. Despite containing all known oviduct-specific regulatory elements, the ectopic expression of the transgene was detected in non-oviduct tissues of the chimeric chicken when utilizing these regions. [7]. In other studies, the estrogen-responsive enhancer element (ERE, located approximately 3.3 kb upstream of the transcription start site in the genome [35]) was incorporated into the construct containing the *OVA* promoter [8, 9]. However, the results of the study did not demonstrate any increase in the level of recombinant protein produced in transgenic chickens [8]. Herron et al. reintroduced an additional regulatory sequence between the ERE and SDRE elements in their targeting construct to enhance the expression level of protein in the egg white [15]. The *OVA* promoter, ranging from 1.35 kb to 3.0 kb in length, which has been used in most of the *ex-situ* studies so far, contains five main conserved sites that have been identified in chicken and other avian species [36]. These studies were unable to evaluate the functions of these regulatory elements within a genomic context, where additional factors such as trans-acting regulatory elements, the chromosomal structure of the gene locus, and the three-dimensional (3D) nuclear organization [37] are involved.

The experimental work presented here provides the first evaluation of *OVA* regulatory elements within their genomic context, where the trans-acting regulatory elements can exert their effects, leading to the *OVA* gene upregulation. As previously shown by Dougherty et al. [20], the repressor activity associated with the CAR site is mediated by the binding of interferon regulatory factors to this site. Based on this knowledge, we reasoned that binding of dCas9 to the negative regulatory regions would hinder the binding of proteins to the CAR and Silencer regions. Consequently, in the absence of these proteins, the negative regulatory sequences would not be able to exert their inhibitory effect on the expression of the *OVA* gene. Therefore, we hypothesized that the spatial occupancy of the CAR and silencer regions may serve as a physical barrier, preventing the binding proteins to access these sites.. Using the DF1 fibroblast cell line, we first showed that CRISPR interference (CRISPRi) exerted on certain regulatory elements of the promoter results in the upregulation of the *OVA* expression. Second, by deleting the *OVA* distal promoter elements including SDRE and NRE via dual sgRNA CRISPR/Cas9-mediated excision, we observed an increased expression of the *OVA* gene. Finally, we evaluated the activity of a foreign gene within this modified region by integrating a transgene reporter under the control of the engineered promoter via CRISPR HDR (homology-directed repair). Our findings indicate that the targeted modification and engineering of the promoter have led to a significant upregulation of the *OVA* gene in the absence of estrogen activation. The methodology applied here overcomes the limitation of cloned promoters, where the promoter regulatory sequences have to be taken out of their native spatial nuclear organization into a plasmid for further evaluation.

## Results

### CRISPR interference of the regulatory sequences in the *Ovalbumin* promoter

**Fig. 1:**
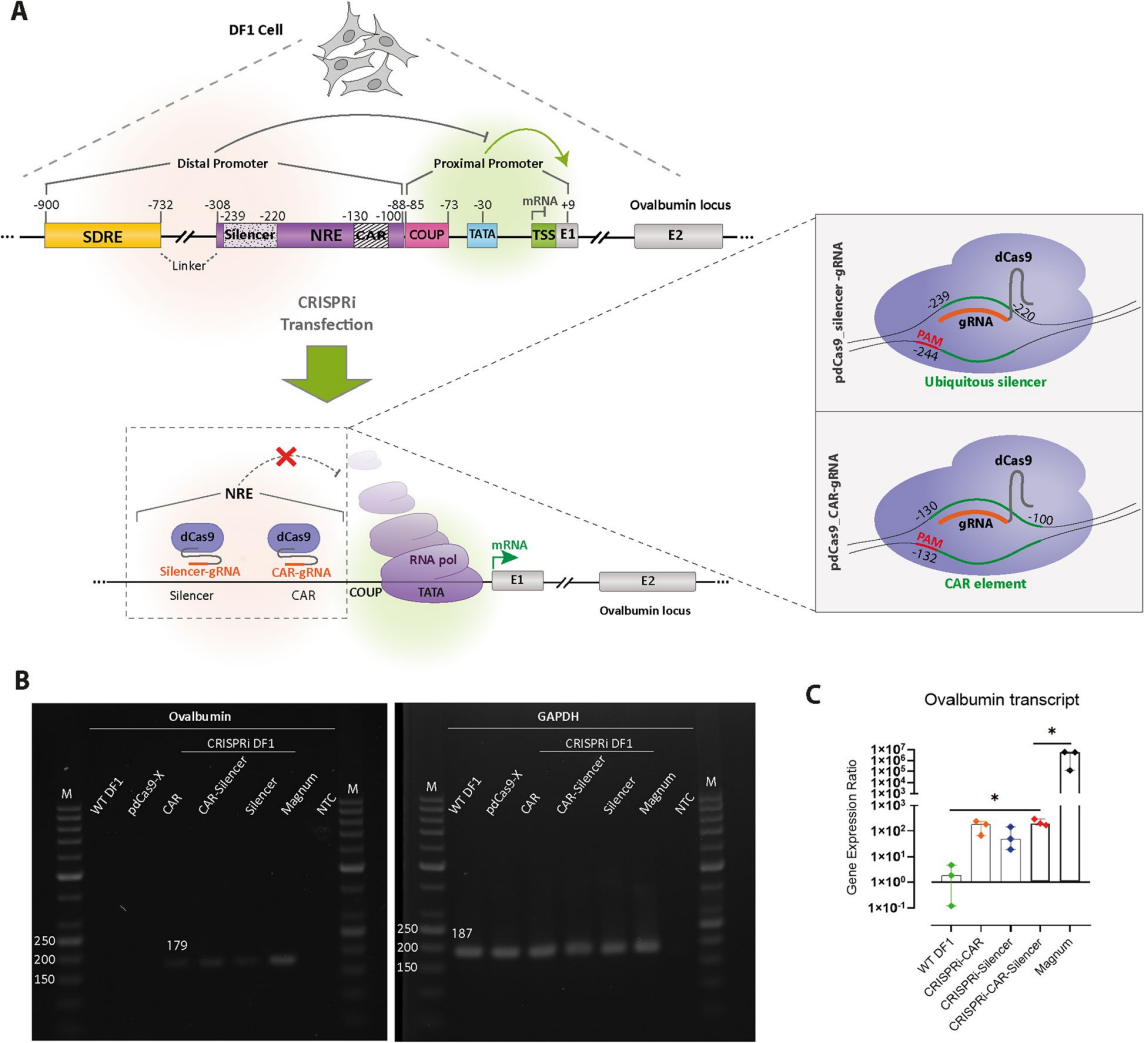
CRISPRi-mediated activation of the *Ovalbumin* promoter in DF1 cells. **A** The schematic representation of the promoter and coding region of the *OVA* gene in DF1 cells. Two regulatory elements of SDRE and NRE are shown in the distal promoter. The bottom panel shows binding sites for two guide RNAs (Silencer-gRNA and CAR-gRNA) that bind the silencer and CAR regions in the NRE element, respectively. SDRE, steroid-dependent regulatory element; NRE, negative regulatory element; CAR, COUP-adjacent repressor site; COUP, Chicken *OVA* upstream promoter; TATA, TATA box; TSS, transcription start site; dCas9, Catalytically dead Cas9. The enlarged inset in the lower section of panel A shows the location and orientation of PAM regions and protospacers for the two regulatory regions of ‘silencer’ and ‘CAR’. **B** The left panel shows agarose gel electrophoresis for analysis of the RT-PCR products which were amplified by primers P8 and P9 (for *OVA*, Fig. 2A and Table 1). The right panel shows agarose gel electrophoresis for analysis of the RT-PCR products which were amplified by P10 and P11 (for *GAPDH*, Table 1). RNA was extracted from DF1 cells which were transfected with CRISPRi vectors that target the NRE element at CAR, Silencer, both CAR and silencer sequences, and pdCas9-X as the negative control. The expected amplicon size for *OVA* was 179 bp, and for *GAPDH* was 187 bp. WT, wild-type; Magnum, hormonally-activated tissue of magnum from  a 35-week-old laying hen; M, DNA size marker; NTC, no template control. **C** Upregulation of the *OVA* mRNA in CRISPRi-modified DF1 cells was assessed by RT-qPCR. Upon transfection with CRISPRi vectors that target the NRE element at CAR, Silencer, and both CAR and silencer sequences, an increment in the *OVA* gene expression level was determined. The transcript levels for *OVA* in the hormonally-activated tissue of the magnum (from a 35-week-old laying hen) show the highest level of expression. The gene expression ratio for the *OVA* over *GAPDH* was calculated by the Pfaffl method of relative quantification [38]. For each group of CRISPRi-DF1 cells, three biological replicates were used. Each biological replicate was read as three technical replicates. The Mann–Whitney assay was used to analyze significant statistical differences between groups. The asterisk (*) indicates statistical differences with *p* values < 0.05

The previous plasmid-based reporter assays have shown that the SDRE and NRE regulatory elements are important for the promoter activity of the *OVA* gene. Deletion of these two elements, as well as the linker in between, results in an increased reporter gene activity in an estrogen-independent manner [24, 27, 28]. We hypothesized that it is the negative effects of the NRE element in the distal promoter that keep the *OVA* gene transcriptionally inactive in the absence of estrogen in non-oviduct cells (Fig. 1A). We transfected DF1 fibroblast cells with plasmids encoding dCas9, as well as CAR and silencer sgRNAs, which targeted the CAR, and silencer sequences of the NRE element, respectively (Tables 1 and 2). Three days after transfection, we were able to detect the transcription of the *OVA* gene (Fig. 1B), while transfection of dCas9 without sgRNA (pdCas9-X) did not result in transcriptional activation. Our RT-qPCR results showed that the expression of *OVA* in DF1 fibroblast cells subjected to CRISPR interference with two sgRNAs was more than 100-fold and significantly higher (*p* < 0.05) than that in wild-type DF1 cells (Fig. 1C and S1). These experiments indicated that the negative effects of the NRE element on the *OVA* gene can be counteracted by CRISPR interference. We reasoned that one possible mechanism for the negative effects of the NRE element on the transcription of the *OVA* gene could be exerted by regulatory RNAs originating from the distal promoter. However, using PCR or hemi-nested PCR, we were not able to identify any RNA transcripts that might originate from the NRE element (Fig. S2, Table 1).

**Table 1:** Oligonucleotides used in this study

**Gene**	**Sequence (5' to 3')**	**Product (bp)**	**Application**
	F: CACCGgttggatgggagagaagact R: AAACagtcttctctcccatccaacc	-	Silencer-gRNA (Targeting silencer)
	F: CACCGattgaaactaaaatctaacc R: AAACggttagattttagtttcaatc	-	CAR-gRNA (Targeting CAR)
	P1: caccgtcctgatggattagcagaac P2: cccagaattaaaaactaatatttgctctcc P3: caccggctctccattcaatccaaaa P4: ccatagaaatcatttaatgggattggg	(P1 and P4):140 bp (P2 and P4):95 bp (P3 and P4):77 bp	RT-PCR (Detection of Non-coding RNAs)
*Ovalbumin* (J00895) and its promoter	F: CCCAAGCTTGGGTCGCG ATCGCGAcctctgctttctcatatatctgtcc R: CGGATATCCGGCCCCTTA AGGGGtagagctgacatgatggcaayg	556	Cloning of the left homology arm
	F: CCCAAGCTTGGGCTAGC TAGCTAggtgcaaaagacagcaccaggac R: CCGGATATCCGgtttgttctgaat cccctgttacttcc	526	Cloning of the right homology arm
	F: CACCGaatgatttctatggcgtcaa R: AAACttgacgccatagaaatcattc	-	NRE-gRNA (Targeting downstream of CAR^a^)
	F: CACCGtaaacttcagctagtggtat R: AAACataccactagctgaagtttac	-	SDRE-gRNA (Targeting upstream of SDRE^b^)
	F: CACCGgctctagccatggtatacct R: AAACaggtataccatggctagagc	-	*OVA* E2-gRNA (Targeting *Ovalbumin* exon 2)
	P5: aatattatttgcactaccatcttgtct P6: gtgcaagtaagagctaatatagagag P7: cacccttaaagatacaacacatagcaca	WT^c^ (P5 and P7):1310 DEL^d^ (P5 and P7): ~ 370 WT (P5 and P6):1256 DEL (P5 and P6): ~ 316	Genomic PCR confirmation of promoter deletion
*Ovalbumin* (NM_205152)	P8: tgctgttgcctgatgaagtctc P9: aatgcccatagccattaagacaga	179	RT-qPCR
*GAPDH* (NM_204305)	P10: cagaacatcatcccagcgtcc P11: cagcagccttcactaccctc	187	RT-qPCR
*Ovalbumin* and Dsred2	P12: taccttctctctatattagctctta P13: ggtgcttcacgtacaccttg	~ 2569	Genomic PCR confirmation of transgene integration

^a^*CAR* COUP-adjacent repressor (CAR) site (− 130 to − 100) in the negative regulatory element (NRE; − 308 to − 88)

^b^*SDRE* steroid-dependent regulatory element (− 900 to − 732)

^c^*WT* wild-type promoter

^d^*DEL* promoter with a deleted region

**Table 2:** DNA constructs used in this study

Construct	Features	Application
pdCas9_silencer -gRNA	hU6 promoter- Silencer_gRNA -sgRNA scaffold-CAG promoter-dCas9-polyA	CRISPRi
pdCas9_CAR-gRNA	hU6 promoter- CAR_gRNA -sgRNA scaffold-CAG promoter-dCas9-polyA	CRISPRi
pdCas9-X	CAG promoter-dCas9-polyA	CRISPRi control
pX459_14	hU6 promoter- NRE_gRNA -sgRNA scaffold-CAG promoter-Cas9-T2A- PuroR-bGH polyA	CRISPR Excision
pX459_15	hU6 promoter- SDRE_gRNA -sgRNA scaffold-CAG promoter-Cas9-T2A- PuroR-bGH polyA	CRISPR Excision
pX459_6	hU6 promoter- *Ova* E2_gRNA -sgRNA scaffold-CAG promoter-Cas9-T2A- PuroR-bGH polyA	CRISPR HDR
pHD_4520	Exon 2 LHA-IRES-DsRed2-HSV TK polyA-CMV promoter –PuroR-IRES2-EGFP-SV40 polyA-Exon 2 RHA	CRISPR HDR

*hU6* human U6 promoter, *sgRNA* single guide RNA, *PuroR* puromycin N-acetyltransferase resistance gene, *bGH polyA* bovine growth hormone polyadenylation signal, *SV40 polyA* SV40 polyadenylation signal, *IRES* internal ribosome entry site, *CAR* COUP-adjacent repressor site (− 130 to − 100) in the negative regulatory element (NRE; − 308 to − 88), *SDRE* steroid-dependent regulatory element (− 900 to − 732), *LHA* left homology arm, *RHA* right homology arm

### Deletion of the distal elements in the *Ovalbumin* gene promoter induces the expression of the *Ovalbumin* mRNA

**Fig. 2:**
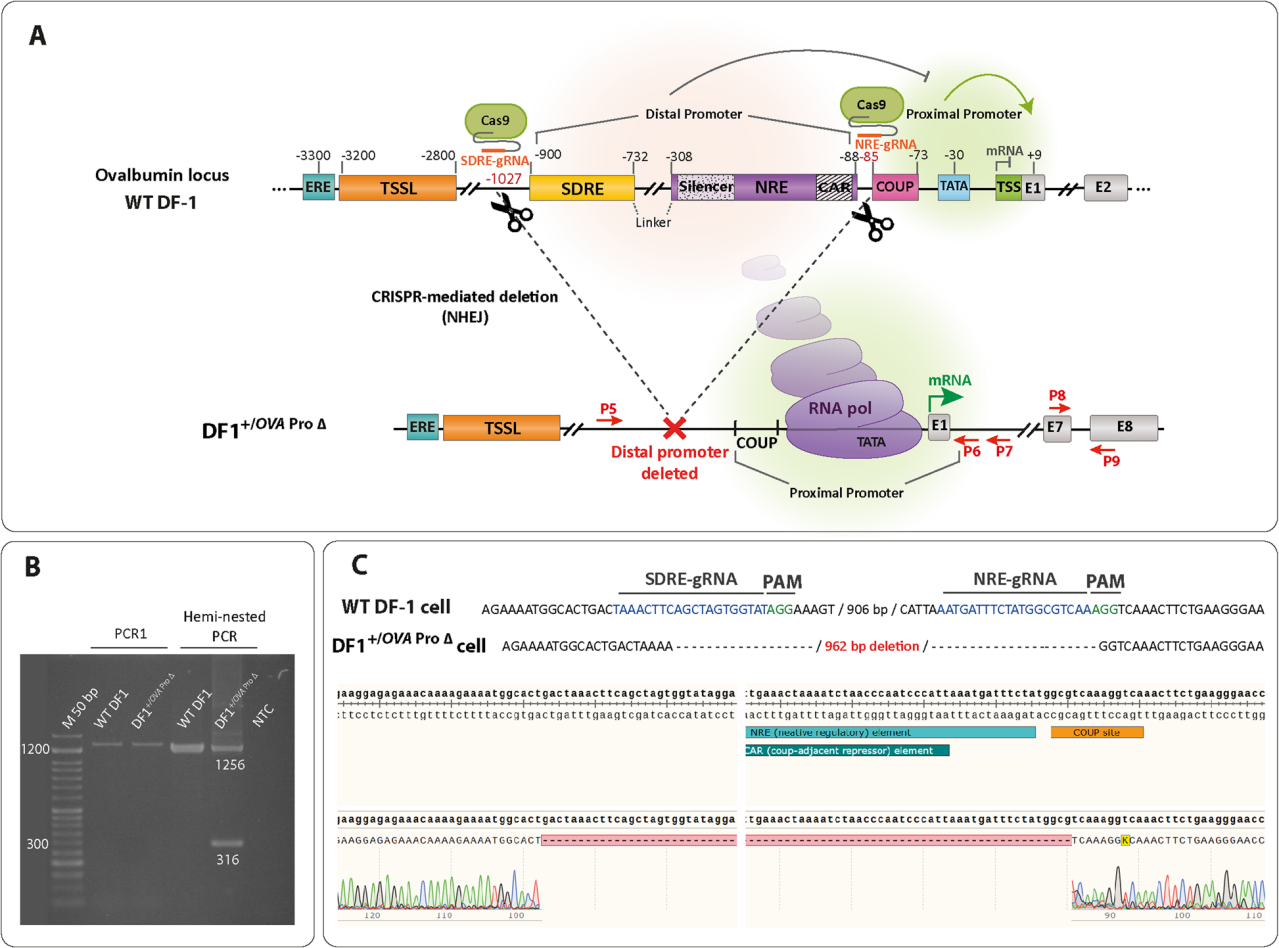
Design and validation of the targeted deletion of *Ovalbumin* distal promoter elements in DF1 cells. **A** The schematic representation of CRISPR/Cas9 mediated deletion strategy of the *OVA* promoter in DF1 cells. The top diagram shows the wild-type (WT) chicken *OVA* locus. The two guide RNA (SDRE-gRNA and NRE-gRNA) binding sites are shown. The NRE- and SDRE- gRNAs target two positions downstream of NRE (downstream of CAR) and upstream of SDRE, respectively. The bottom diagram shows the locus after CRISPR-mediated deletion of the distal *OVA* promoter in DF1 cells (DF1^+/*OVA* Pro ∆ cell^). The PCR primers used for the assessment of deletion (P5 to P7), and the *OVA* gene expression (P8 and P9, used in Figs. 1 and 3) are shown as small red arrows. **B** Two-step genomic PCR to confirm the deletion of the distal promoter of the *OVA* gene. In the first PCR (using P5 and P7 primers, Table 1), an amplicon of 1310 bp was amplified from the wild-type (WT) allele (In the first PCR, amplicon of ~ 370 bp were not detected from the promoter-deleted (DF1^∆^) alleles). In a hemi-nested PCR (using P5 and P6 primers), amplicons of 1256 bp and ~ 316 bp were amplified from the wild-type and promoter-deleted (DF1^∆^) alleles, respectively. **C** Alignment of the representative sequences of the wild-type (WT DF1) and promoter-deleted (DF1^∆^) sequences determined by Sanger sequencing. The gRNA-binding sites are shown in blue, and the PAM regions are shown in green letters. WT, wild-type; DF1 ^∆^, DF1 cells knockout for the distal *OVA* promoter (DF1 ^+/*OVA* Pro ∆^); NHEJ, non-homologous end-joining; ERE, estrogen-responsive enhancer element; TSSL, tissue-specific silencer-like element; SDRE, steroid-dependent regulatory element; NRE, negative regulatory element; CAR, COUP-adjacent repressor site; COUP, Chicken *OVA* upstream promoter; TATA, TATA box; TSS, transcription start site; P, primer. M, DNA size marker; NTC, no template control

An alternative mechanism for the effects of the distal promoter on gene transcription could be mediated by intra-chromosomal contacts (loops) that bring together the distal regulatory segments to the core promoter [37]. Previous studies have shown that the cloned proximal segment of the *OVA* promoter lacking the major regulatory elements of SDRE and NRE, can significantly increase (up to 17-fold) the chloramphenicol acetyltransferase (CAT) gene activity on a plasmid construct in LMH cells (a chicken hepatoma cell line) and chicken primary oviduct cells, and this increase occurs in an estrogen-independent manner [27, 29]. Thus, we asked whether the deletion of the SDRE and NRE elements from the native promoter would be able to increase the transcription of the *OVA* gene in a non-oviduct cell. To this end, we used the CRISPR excision strategy to delete the SDRE and NRE elements from the *OVA* promoter in DF1 fibroblast cells (Fig. 2A). To confirm this deletion, these cells (DF1 ^+/*OVA* Pro ∆^) were subjected to genomic PCR and Sanger sequencing (Figs. 2B and C). Then, individual cells were grown in three 96 well plates to acquire correctly edited isogenic clones for subsequent expansion and validation of gene expression.

**Fig. 3:**
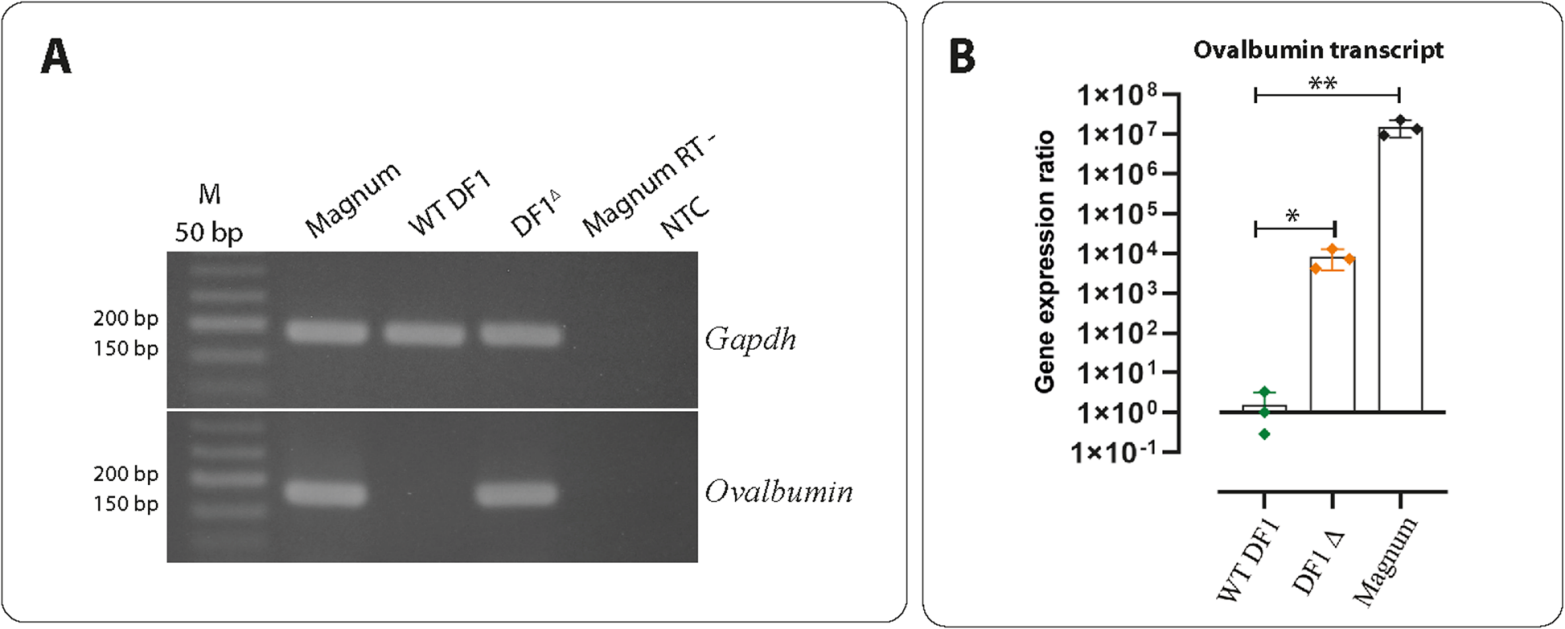
Gene expression ratio for *Ovalbumin* transcript in DF1^+/*OVA* Pro ∆^ cells. **A** Agarose gel (2%) electrophoresis for analysis of the RT-PCR products amplified by primers P8 and P9 (for *OVA*, Fig. 1), and P10 and P11 (for *GAPDH*). The expected amplicon size for *OVA* and *GAPDH* are 179 bp and 187 bp, respectively. WT, wild-type; DF1 ^∆^, distal *OVA* promoter knockout DF1 cells (DF1 ^+/*OVA* Pro ∆^); M, DNA size marker; NTC, no template control; RT, reverse transcriptase. The full-length gel electrophoresis images are shown in Fig. S3. **B** Upregulation of the *OVA* mRNA in DF1 ^+/*OVA* Pro ∆^cells was assessed by RT-qPCR. Upon deletion of the distal *OVA* promoter, an increased level of expression of the *OVA* gene was determined (DF1^∆^). The transcript levels of *OVA* for these samples (Three isogenic DF1 ^+/*OVA* Pro ∆^ clones) were ~ 10^4^-fold higher than the *OVA* transcript levels in the wild-type DF1 (WT DF1). The transcript levels for *OVA* in the hormonally-activated tissue of the magnum (from a 35-week-old laying hen) show the highest level of expression. The gene expression ratio for the *OVA* over *GAPDH* was calculated by the Pfaffl method of relative quantification [38]. The Mann–Whitney assay was used to analyze significant statistical differences between the WT-DF1 group and DF1^∆^ and magnum groups. * and ** show statistical differences with *p* values < 0.05 and < 0.01, respectively

Three isogenic DF1 ^+/*OVA* Pro ∆^ clones with the confirmed deletion of SDRE and NRE elements were cultured in vitro without estrogen and were analyzed for the expression of the *OVA* gene by RT-qPCR. The transcript levels of the *OVA* gene in the DF1 ^+/*OVA* Pro ∆^ cells increased more than 10^4^-fold compared to that in the wild-type DF1 cell (*p* < 0.01) (Fig. 3). The transcript levels of the *OVA* gene in the hormonally-activated tissue of the magnum from the 35-week-old laying hen were 10^7^-fold higher compared to that in the wild-type DF1 cells (Figs. 3A and B, Fig. S3, Table 1).

### A fluorescent genomic reporter is activated under the control of the *Ovalbumin* promoter with the deletion of distal elements

**Fig. 4:**
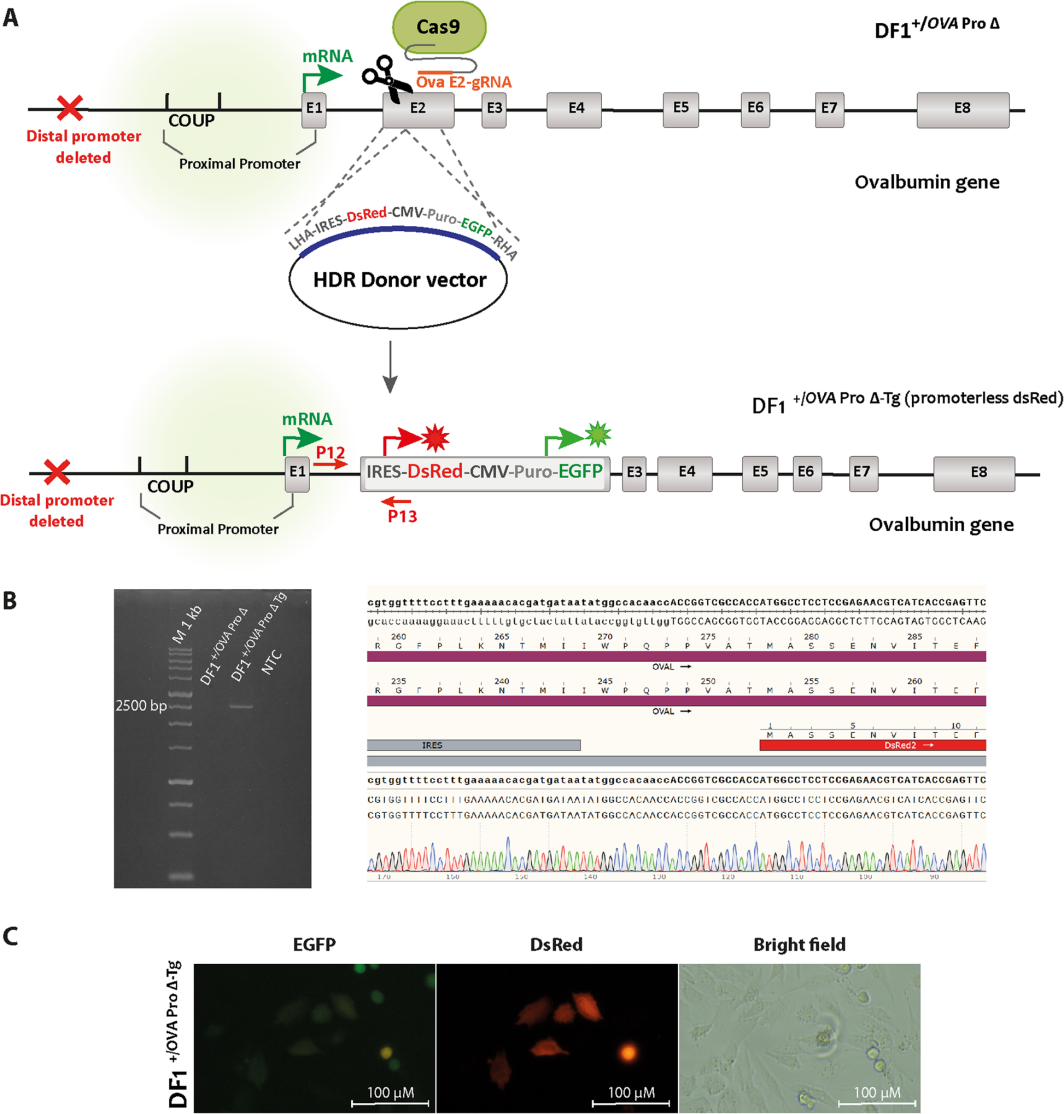
Activation of transgene expression in DF1 ^+/*OVA* Pro ∆−Tg (promoterless dsRed)^ cells. **A** The schematic representation of CRISPR HDR mediated knockin strategy in DF1^+/*OVA* Pro ∆^ cells. The top diagram shows the donor vector that was designed to have a promoterless DsRed2 and a CMV-Puro-EGFP cassette flanked by left and right homology arms. The *OVA* E2 indicates the gRNA-binding site on exon 2 of the *OVA* (+ 174 to + 1784) gene. The bottom diagram shows the allele after CRISPR-HDR insertion of the reporter cassette (DF1 ^+/*OVA* Pro ∆−Tg (promoterless dsRed)^). PCR primers (P12 and P13) were used for the assessment of the CRISPR-HDR insertion of the promoterless DsRed2 in DF1 ^+/*OVA* Pro ∆−Tg (promoterless dsRed)^ cells. **B** Genomic PCR analysis of the targeted gene knock-in DF1 ^+/*OVA* Pro ∆−Tg (promoterless dsRed)^ cells. For the assessment of the CRISPR-HDR insertion of the promoterless DsRed2 in DF1 ^∆−Tg^ cells, primers (P12 and P13) were used to amplify a 2569 bp amplicon. The insertion-specific PCR products of DF1 ^∆−Tg^ cells were sequenced by Sanger sequencing and aligned to the donor plasmid (used as a DNA repair template during transfection). **C** Fluorescence microscopy of DF1 ^∆−Tg^ cells indicating DsRed2 expression under the control of the endogenous truncated *OVA* promoter, Magnification: 20X. DF1^∆^, DF1 cells knock-out for distal *OVA* promoter (DF1 ^+/*OVA* Pro ∆^); DF1 ^∆−Tg^ cells, promoterless DsRed2 knockin DF1 cells (DF1 ^+/*OVA* Pro ∆−Tg (promoterless dsRed)^); HDR, homology-directed repair; M, DNA size marker; WT, wild-type; NTC, no template control

Next, we asked whether the *OVA* gene promoter with the deletion of its distal elements in the DF1 ^+/*OVA* Pro ∆^ cells can activate a foreign transgene. For this purpose, we designed a reporter construct containing a promoterless DsRed2 (IRES-DsRed2-HSV TK polyA-CMV promoter-PuroR-IRES2-EGFP-SV40 polyA) and inserted it into exon 2 of the *OVA* gene, 125 bp after ATG codon, using CRISPR HDR (Fig. 4A). In these cells (DF1 ^+/*OVA* Pro ∆−Tg (promoterless dsRed)^), the insertion of the reporter was confirmed by genomic PCR, Sanger sequencing, and fluorescence microscopy for GFP (Figs. 4B, C). The promoterless DsRed2 reporter, under the function of a distally-deleted *OVA* promoter, became activated, and its red fluorescence was visualized using fluorescence microscopy (Fig. 4C). However, when the promoterless reporter was inserted at the same region in the *OVA* locus of the wild-type DF1 cells, it did not result in red fluorescence (Fig. S4). This experiment confirmed that non-oviduct chicken cells with the deletion of distal elements in their *OVA* promoter can express an inserted transgene in an estrogen-independent manner. The wild-type DF1 cells did not show any transcriptional activity for the *OVA* gene (Fig. 3).

## Discussion

In this study, we have shown that the negative effects of the NRE element on the *OVA* gene can be counteracted to some extent by CRISPR interference (Fig. 1). We have also demonstrated that the deletion of the distal *OVA* promoter in DF1 cells (DF1 ^+/*OVA* Pro ∆^) leads to the induction of the *OVA* gene expression (Figs. 2 and 3). In addition, the insertion of a promoterless reporter in these cells (DF1 ^+/*OVA* Pro ∆−Tg (promoterless dsRed)^) resulted in the expression of the fluorescent reporter protein (DsRed2) (Fig. 4), indicating that a chicken non-oviduct cell line with the deletion of distal promoter sequences can serve as a model for steroid-independent expression of a transgene driven by the endogenous *OVA* promoter.

The tissue-specific *OVA* promoter has been identified as a novel candidate for the large-scale production of pharmaceutical proteins. It has been effectively employed in the synthesis of several therapeutic proteins [7–17]. Although the regulatory elements in the *OVA* promoter have been fairly well characterized [20–31, 35], it is not clear which regulatory sequences are sufficient and efficient enough to induce oviduct-specific expression of exogenous genes. Previous studies demonstrated that deletion of the SDRE and NRE, along with the linker between them, increased chloramphenicol acetyltransferase (CAT) activity on a plasmid [24, 27, 28]. These studies indicated that the cooperation between multiple distal regulatory and promoter-proximal regions confers oviduct-specific *OVA* expression. Deletion of regulatory elements upstream of − 80 abolished the tissue-specific expression of *OVA* in primary oviduct cell cultures, while basal expression was increased to levels comparable to those seen after estrogen induction in genes that contain an SDRE [24, 27, 30]. Additionally, a few reports have shown that the expression of the reporter CAT gene was induced by the *OVA* proximal promoter (− 87 to + 9) in primary oviduct cells and non-oviduct cell cultures, such as LMH/2A (Table 3) [25, 27–30, 39, 40].

**Table 3:** A summary of findings on the analysis of promoter regulatory regions of the chicken *Ovalbumin* gene

	**Schweers, 1990**	**Haecker, 1995**	**Dean, 1996**	**Muramatsu, 1998**	**Sensenbaugh, 1999**	**Park, 2000**	**Monroe, 2000**	**This study**
Strategy	In vitro gene transfer of OvCAT fusion genes containing mutations in the SDRE and NRE	In vitro gene transfer of OvCAT fusion genes containing truncated O*valbumin* promoter with various lengths of the NRE and mutation in the silencer	In vitro gene transfer of OvCAT fusion genes containing mutations in the SDRE	In vivo gene transfer of OvCAT fusion genes containing various lengths of truncated O*valbumin* promoter	In vitro gene transfer of OvCAT fusion genes containing mutations in the SDRE	In vitro gene transfer of OvCAT fusion genes containing truncated Ovalbumin with deletions in the NRE, the SDRE, and mutations in the COUP, and overexpression of COUP-TF1	In vitro gene transfer of OvCAT fusion genes containing truncated O*valbumin* promoter with deletions in the NRE, the SDRE, and mutations in the CAR and the silencer	In vitro gene transfer of CRISPR excision for In situ deletion of the genomic SDRE/NRE and CRISPR HDR for insertion of promoter-less reporter
Cell type	Primary oviduct cells	Primary oviduct cells	Primary oviduct cells	Oviduct and liver of laying hens	Primary oviduct cells	Primary oviduct cells	LMH/2A cell line	DF1 cell line
Findings	Induction of the *Ovalbumin* gene by steroid hormones requires complex interactions involving both the SDRE and NRE	The NRE is a multifunctional regulatory element containing at least two sites for induction by steroids and three elements that repress *Ovalbumin* transcription	The *Ovalbumin* gene is regulated by steroid Hormones, binding to a DNA element from -891 to -878 in the SDRE	The *Ovalbumin* gene promoter region between -3200 and -2800 bp (a tissue-specific silencer-like) represses the *Ovalbumin* gene transcription in the liver, but not in the oviduct of laying hens	The NRE contains not only the sites responsible for the repression of the gene but also a positive element that is required for the responsiveness to steroid hormones	Without the NRE, the SDRE is sufficient for induction by estrogen, irrespective of the COUP site. with the NRE intact, the COUP site is required for steroid induction. Without the NRE, the COUP site attenuates transcriptional activity	The inhibition of *Ovalbumin* gene expression in non-oviduct cells is a combination of the lack of essential positive factors and the presence of an active repressor,which binds to the CAR element	In situ genomic deletion of the SDRE and NRE is sufficient to derepress the transcription of the *Ovalbumin* gene and induced the activity of an inserted transgene in the non-oviduct cells
Comment	Although previous studies have provided insights into the mechanisms that underlie the hormonal, and tissue-specific regulation of *Ovalbumin* gene expression, most have applied plasmid-based methods, irrespective of the genome context. Combining genomics or transcriptomics approaches with plasmid-based MPRA (massively parallel reporter assays) and CRISPR-based in vivo methods can develop our understanding of the mechanisms underlying regulatory events of gene expression. In this study, to consider the genomic context, we have applied CRISPR tools to manipulate the genomic regulatory regions of the *Ovalbumin* promoter							

*OvCAT Ovalbumin* promoter driving CAT (chloramphenicol acetyltransferase) reporter, *SDRE* steroid-dependent regulatory element, *NRE* negative regulatory element

**Fig. 5:**
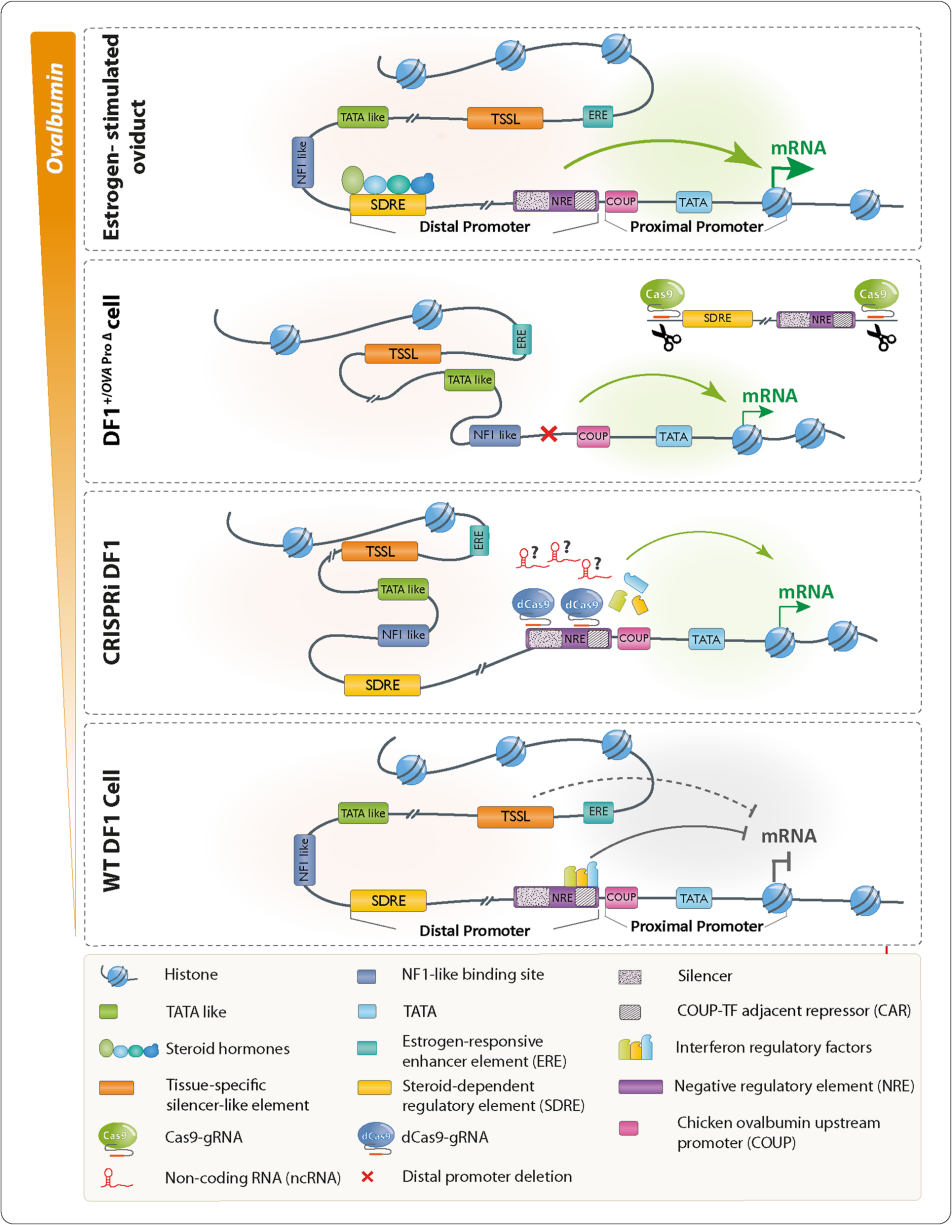
A schematic model depicting the mechanism of increased expression of the *Ovalbumin* gene in different cell types in steroid-dependent and –independent manners. The main induction for the expression of the *OVA* gene in oviduct cells is estrogen that by binding to the SDRE region overcomes the inhibitory circuits exerted by the tissue-specific silencer-like element (TSSL), and negative regulatory element (NRE). The CRISPR/CAS-mediated deletion of the regulatory sequences of the *OVA* distal promoter (SDRE, NRE, and the linker in between) leads to the expression of the *OVA* gene in DF1 cells. The CRISPR-mediated interference of regulatory sequences of the NRE element as well leads to an increased expression of the *OVA* gene in DF1 cells

In this investigation, we studied the role of regulatory elements of the *OVA* promoter in their natural genomic context. CRISPRi was performed on two regulatory sequences of the NRE element, CAR and silencer, using dCas9 and manually selected sgRNAs (Fig. 1A). This resulted in a likely counteracting effect exerted on the NRE element, which, in turn, increased the transcription of the *OVA* RNA (Fig. 1B). Researchers have used various strategies including biochemical methods [41], crystallographic methods [41, 42], and atomic force microscopy [43] to identify the length of Cas9/dCas9 footprint on the DNA template. Zhang et al., [44] used a single-molecule approach to measure the footprint and determined the length of the DNA over which Cas9 binding likely affects the binding of another protein. To ensure interference of binding proteins with CAR and Silencer, we manually selected sgRNAs that specifically target these sites. Our CRISPR interference experiment confirmed the negative role of both CAR and silencer in *OVA* gene expression. The expression of *OVA* subjected to CRISPR interference with two sgRNAs was significantly higher and more than 100-fold (*p* < 0.05) than that in the wild-type DF1 cells. In the next set of experiments, we decided to knock out these regulatory sequences to examine their potential impact on the transcription of the *OVA* gene. Our findings demonstrated that the in situ deletions of the distal *OVA* promoter led to the upregulation of *OVA* transcript in DF1 cells. Our RT-qPCR analysis, following the deletion of the distal *OVA* promoter which includes the SDRE and the NRE, in the DF1^+/*OVA* Pro ∆^ cells, revealed a significant increase of approximately10^4^-fold in *OVA* transcript levels compared to wild-type DF1 cells (Fig. 3). This finding strongly supports our hypothesis that negative regulatory elements have a highly effective role in controlling *OVA* expression. Furthermore, based on the same results, the magnum tissue exhibited transcript levels approximately 10^3^-fold higher than the DF1^+/*OVA* Pro ∆^ cells, indicating that positive regulatory signals, including estrogen, can further boost the expression. We found that the deletion of a 962-bp region (− 1044 to − 82 bp) containing the distal promoter elements resulted in a significant reduction in the tissue-restricted and hormone-dependent expression of the *OVA* gene. It has been reported that the chicken *OVA* upstream promoter (COUP) site (− 85 to − 73) represses basal *OVA* expression in the absence of steroids and is required for its induction by steroids [30]. Although previous reports have shown that the deletion of the COUP site in OvCAT constructs increases transcriptional activity in the absence of the NRE and confirms its repressive role on basal gene expression, our data clearly show that even in the presence of the COUP site, transcriptional activity is increased when the NRE is absent. Muramatsu et al. demonstrated that the sequence from − 3200 to − 2800 acts as a tissue-specific silencer-like (TSSL) element, repressing the expression of *OVA* gene in non-oviduct tissue [40]. Although our experiment with DF1^+/*OVA* Pro ∆^ cells did not detect the effect of TSSL element in repressing the *OVA* gene expression, it remains unclear whether this TSSL element causes tissue-specific repression in other tissues or if universal transcription factors bind to it in all tissues except the oviduct. This finding suggests that the opposing effect of the COUP site on transcriptional activity depends on the native genomic context and, perhaps, other regulatory elements are brought together in a spatial configuration by chromatin loops (Fig. 5).

In our DF1^+/*OVA* Pro ∆^ cells, although the core promoter elements (TATA box and the initiator element, INR) that contain sufficient information for the initiation of transcription have remained intact, we cannot rule out the potential regulatory role of alternative promoters in the genome [45]. Kodama et al. identified several TATA-like and other promoter motifs located at a position around − 1800 bp [10]. Bradshaw et al. demonstrated that the region from − 1094 to − 1125 (− 1100), in the presence of an NF-1-like protein, functions as a steroid hormone-independent enhancer and increases *OVA* gene transcription [46]. A nuclear factor-1-like factor binds to a far upstream *OVA* enhancer [46]. Our results support the notion that the transcriptional regulation of the *OVA* gene is not determined only by promoter regions, but may involve multiple regulatory elements in the local genomic context that work in the three-dimensional organization of the locus [47, 48] (Fig. 5). This three-dimensional organization of the *OVA* locus in the oviduct cells, which might be dependent on the nuclear positioning of chromosomes and/or the architecture of chromatin within chromosome territory [37] can establish a structural scaffold for interaction between enhancer-promoter, enhancer-enhancer, promoter-promoter, and superenhancer elements. These kinds of interactions may be further promoted and changed by the activity of specific transcription factors, signaling pathways, hormones, and developmental stages [49–53]. The overall output from these interactions might result in the transcriptional activation of the *OVA* locus. We hypothesize that in non-oviduct differentiated cells, a specific repressive chromatin organization is established as well, which is perturbed by CRISPRi and the excision of the distal promoter using CRISPR-Cas9 (Fig. 5), leading to the upregulation of the *OVA* gene.

## Conclusions

Our study overcomes the limitation of previous studies that relied on cloned promoters, where the promoter regulatory sequences have to be taken out of their *cis* context and spatial organization into a plasmid. The utilization of CRISPR technology enabled us to precisely interfere with and delete the negative regulatory sequences of the *OVA* gene promoter directly within the chicken cell's native genomic context. We demonstrate that the expression of a transgene can be driven in a hormonally independent manner through the function of the *OVA* gene promoter and associated endogenous regulatory elements.

## Methods

### Plasmid construction

The CRISPR design tool (http://crispr.mit.edu/) was employed to identify sgRNA binding sites within the *OVA* promoter and coding region for the deletion and insertion of the distal promoter and reporter gene, respectively. However, for our CRISPR interference strategy, we manually selected the sgRNAs and analyzed them regarding their off-target binding and secondary structure. Different plasmids were constructed using routine subcloning techniques. To perform CRISPRi, three plasmids were created: pdCas9_silencer-gRNA (encoding sgRNA for targeting the − 241 to − 222 region), pdCas9_CAR-gRNA (encoding sgRNA for targeting the − 129 to − 110 region), and pdCas9-X (with no sgRNA) (Table 2). The CRISPRi vectors were generated by modifying the plasmid pdCas9-DNMT3A-EGFP (#71,666) using standard techniques. The plasmid was digested with BamHI and BsrGI to remove a 1726 bp fragment that included the DNMT3A catalytic domain. The resulting overhangs were filled in, and the plasmid was self-ligated using T4 DNA ligase, resulting in a plasmid named 71666delta. The CAR- and Silencer-gRNA were then subcloned into the BbsI-digested region of this plasmid for CRISPRi.

For CRISPR excision, two plasmids (px459-14 & px459-15) were constructed, expressing Cas9 and sgRNAs targeting the *OVA* distal promoter. The designed sgRNAs were subcloned into the BbsI-digested region of the px459 plasmid for this purpose. To perform CRISPR HDR, another plasmid named px459-6 was created. It contained Cas9 and a sgRNA targeting the *OVA* exon 2. The designed sgRNA was also subcloned into the BbsI-digested region of the px459 plasmid.

The donor vector (pHD_4520) was generated by ligating a 556 bp fragment of the *OVA* gene (beginning of exon 2) representing the 5' homology arm, and a 526 bp fragment of the *OVA* gene, representing the 3' homology arm. To create the donor vector, an initial base vector containing an EGFP reporter gene and necessary restriction sites for subsequent subcloning was synthesized (Table 2). Detailed plasmid maps displaying the specific components can be found in Fig. S7.

### CRISPR interference of the negative regulatory elements of the *Ovalbumin* gene in cultured DF1 cells

DF1 cells were cultured as recommended by the ATCC. The cells were transfected into four groups: group one was transfected with pdCas9_silencer-gRNA, targeting the silencer; group two was transfected with pdCas9_CAR-gRNA, targeting the CAR; group three was transfected with pdCas9_silencer-gRNA and pdCas9_CAR-gRNA; and the control group was transfected with pdCas9-X (with no sgRNA) (Table 2). Lipofectamine 3000 (Invitrogen, USA) was used for transfections as previously described [54]. Briefly, 0.5 μg from each plasmid was diluted with 50 μl OPTI-MEM + GlutaMax (Thermo Fisher Scientific, USA), mixed with 1 μl Lipofectamine 3000 reagent, and then incubated with 0.1–0.15 × 10^6^ DF1 cells for 4 h. Subsequently, the cells were cultured in 500 μl of an antibiotic-free DMEM-F12 culture medium (Thermo Fisher Scientific) and incubated for 24 h at 38 °C in a 7.5% CO_2_ environment. The medium was replaced with fresh medium containing penicillin and streptomycin antibiotics 24 h after transfection. Transfected cells were passaged for subsequent assays for three days.

The effects of CRISPRi on the expression of the *OVA* gene were analyzed by RT-PCR. From DF1 cells subjected to CRISPRi and the positive control magnum tissue (from a 35-week-old laying hen), total RNA was isolated using the Total RNA Isolation Kit (DENAzist Asia, Iran). After checking the quality and quantity of isolated RNA using gel electrophoresis and a spectrophotometer (Epoch 2, BioTek Instruments Inc., USA), total RNA was reverse transcribed using MMLV reverse transcriptase and random hexamer primer (Thermo Fisher Scientific, USA). The complementary DNA for *OVA* and *GAPDH* transcripts was subjected to PCR amplification using Taq DNA Polymerase 2 × Master Mix RED (Ampliqon, Denmark) and specific primers (Table 1). The amplification steps included an initial 95 °C for 3 min, followed by 35 cycles of 95 °C for 30 s, 58 °C for 30 s, and 72 °C for 10 s, with the final elongation step at 72 °C for 10 min.

To investigate the presence of regulatory RNAs that might transcribe from the distal promoter, different primers (Table 1) were designed and used in the PCR or hemi-nested PCR amplification reactions on cDNA which was generated from wild-type DF1 total RNA.

### Targeted deletion of *Ovalbumin* promoter in cultured DF1 cells

To perform CRISPR excision with dual sgRNAs on the *OVA* promoter, DF1 cells were transfected with pX459-14 and pX459-15 (Table 2) using Lipofectamine 3000 (Invitrogen, USA), The transfectd DF1 cells were exposed to puromycin dihydrochloride (2.5 μg/ml; Sigma-Aldrich, USA) for 3 days. DF1 cells after antibiotic exposure were expanded for 2 to 3 weeks. A mixed population of these cells was initially screened using genomic PCR to confirm the deletion of the *OVA* distal promoter in a fraction of cells. Genomic DNA was extracted from wild-type and knockout DF1 cells (DF1 ^+/*OVA* Pro ∆^) using the Genomic DNA Extraction Kit (DENAzist Asia Co., Iran). Gene-targeting events were detected by a single-round or nested PCR using the designed primers (Table 1) and Taq DNA polymerase master mix RED (Ampliqon, Denmark), and confirmed by Sanger sequencing of the amplicons (Genomin Co., Iran). After single-cell isolation and clonal expansion, three clones of knockout DF1 cells with the deletion of the distal *OVA* promoter (DF1 ^+/*OVA* Pro ∆^) were confirmed using genomic PCR. These three clones were analyzed for the expression of *OVA* by RT-qPCR.

### Analysis of *Ovalbumin* expression in DF1 cells with the deletion of distal *Ovalbumin* promoter

Total RNA was isolated from the magnum tissue (from a 35-week-old laying hen), wild-type DF1 cells and DF1 cells knockout for distal *OVA* promoter (DF1 ^+/*OVA* Pro ∆^) using the Total RNA Isolation Kit (DENAzist Asia, Iran). Total RNA was subjected to quality and quantity analysis, and reverse transcription using MMLV reverse transcriptase and random hexamer primers (Thermo Fisher Scientific, USA). Each quantitative PCR reaction contained 1 × SYBR Green Real-time PCR Master Mix (Thermo Scientific, USA), 2μl cDNA template, and each primer (Table 1) at 500nM in a 20μl reaction volume, which was performed in a Rotor-Gene Q real-time PCR cycler (Qiagen, USA). To amplify complementary DNA for *OVA* and *GAPDH* transcripts, the amplification steps were: 95 C for 15min, followed by 35 cycles of 95 C for 30 s, 58 C for 30 s, and 72 C for 30s. To acquire melting curves, the temperature was increased in steps of 0.2 C for 5s from 55 °C to 95 C. PCR products after clean-up with the PCR Clean-up Kit (DENAzist Asia Co., Iran), were subjected to Sanger sequencing (Genomin Co., Iran). (Fig. S5).

Different qPCR reactions were performed to adjust the reaction temperature, find the best concentration of primers, and optimize the amplification and melting curves (Fig. S3). Complementary DNA from the magnum of the 35-week-old hen was serially diluted and subjected to qPCR to make standard curves (Fig. S6). Each dilution was subjected to real-time readings in triplicate. To make a standard curve (Fig. S6), the log^10^ of cDNA concentration for the *OVA* and *GAPDH* genes were plotted against the cycle threshold (Ct) numbers. We used the equation of E = (10^–1/slope^-1) × 100% to calculate the reaction efficiency. The gene expression ratio for the *OVA* gene over *GAPDH* was calculated for the magnum, wild-type DF1, and DF1 cell with deletion of distal *OVA* promoter using the Pfaffl method of relative quantification [38].

### Targeted knock-in of a reporter in DF1 cells with the deletion of distal *Ovalbumin* promoter

DF1 ^+/*OVA* Pro ∆^ cells were transfected with pX459_6 and pHD_4520 (donor vector) using Lipofectamine 3000 (Invitrogen, USA), as described above. The cells 48h after transfection were subjected to antibiotic selection with puromycin dihydrochloride (2.5 μg/ml; Sigma-Aldrich, USA). To confirm the knock-in of the reporter construct (DsRed2-CMV-Puro-IRES-EGFP), genomic PCR and Sanger sequencing (Genomin Co., Iran) were performed. Cells with the inserted reporter and deleted *OVA* promoter (DF1 ^+/*OVA* Pro ∆−Tg (promoterless dsRed)^) were observed and photographed by fluorescence microscopy (Nikon Eclipse Ts2R, Japan) two weeks after transfection.

## Supplementary Information

**Additional file 1.** 

**Additional file 2.** 

**Additional file 3.** 

**Additional file 4.** 

**Additional file 5.** 

**Additional file 6.** 

**Additional file 7.** 

## Data Availability

The data supporting the conclusions of this article are included within the article (and its additional files) and are also available from the corresponding author upon request.

## Abbreviations

CRISPR-HDR: CRISPR-mediated homology-directed repair
ERE: Estrogen-responsive enhancer element
SDRE: Steroid-dependent regulatory element
NRE: Negative regulatory element
CAR: COUP-TF adjacent repressor
TSSL: Tissue-specific silencer-like element
COUP: Chicken *Ovalbumin* upstream promoter
CRISPR: Clustered regularly interspaced short palindromic repeats
sgRNA: Small guide RNA
PAM: Protospacer adjacent motif
dCas9: Endonuclease deficient Cas9, Catalytically dead Cas9
*OVA*: *Ovalbumin* Gene
CAT: Chloramphenicol acetyltransferase
CRISPRi: CRISPR interference
OvCAT: Truncated *OVA* promoter-CAT reporter
INR: Initiator element
NHEJ: Non-homologous end-joining
TSS: Transcription start site
NTC: No template control
DF1∆: Promoter-deleted alleles
TATA: TATA box
WT: Wild-type
DF1^+/^^*OVA*^^ Pro ∆ ^cell: Monoallelic CRISPR-mediated deletion of the distal *OVA* promoter in DF1 cells
DF1^+/OVA Pro Δ-Tg (promoterless dsRed)^: Promoterless DsRed2 knockin DF1 cells
