# Supplementary figures and images for: Study of the regulatory elements of the Ovalbumin gene promoter using CRISPR technology in chicken cells
Source: J Biol Eng. 2023 Jul 17;17:46. doi: 10.1186/s13036-023-00367-3 (PMC10353141; doi:10.1186/s13036-023-00367-3)

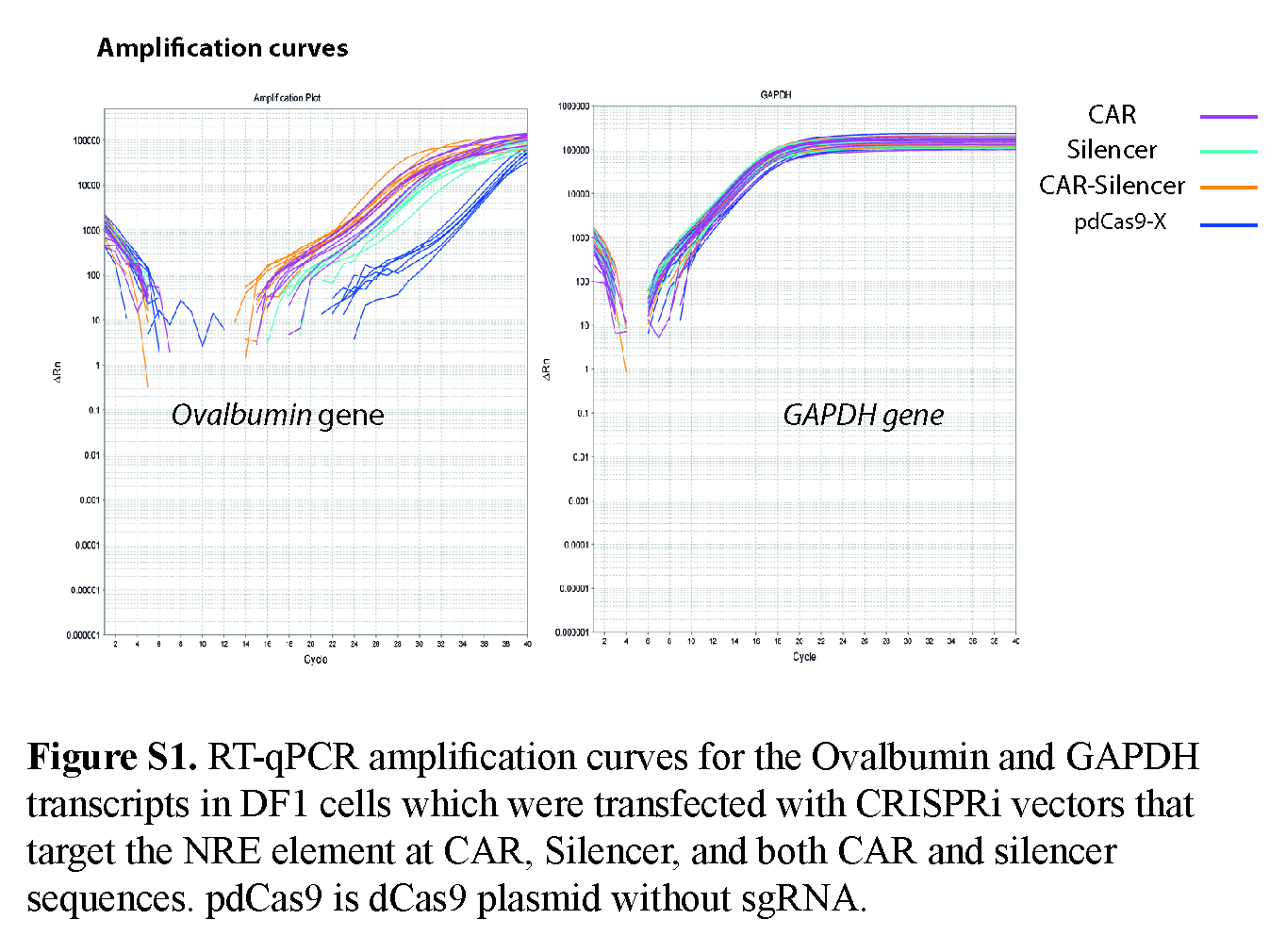

Supplement: Supplementary file 1 — Additional file 1. [file 13036_2023_367_MOESM1_ESM.tif]

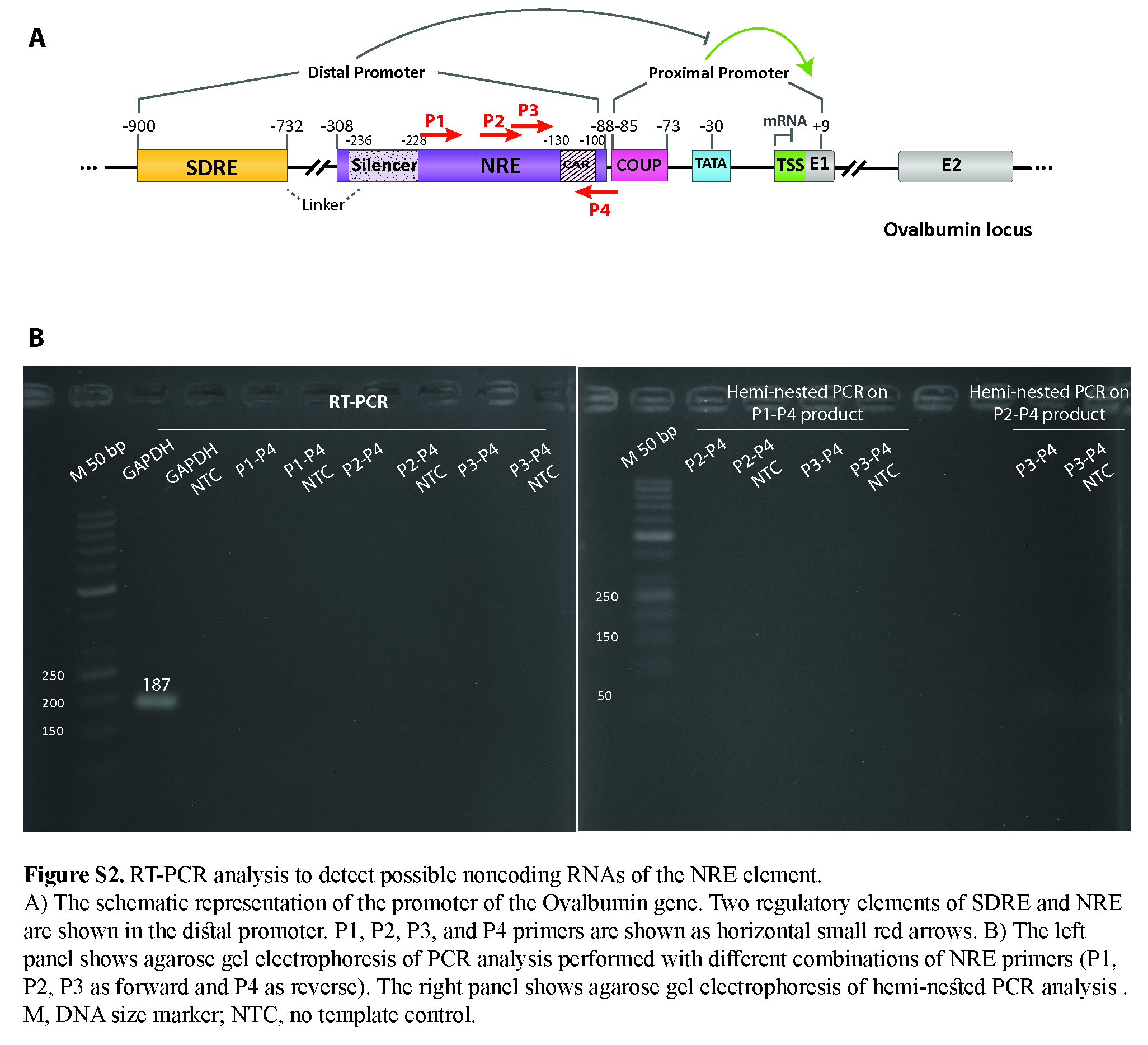

Supplement: Supplementary file 2 — Additional file 2. [file 13036_2023_367_MOESM2_ESM.tif]

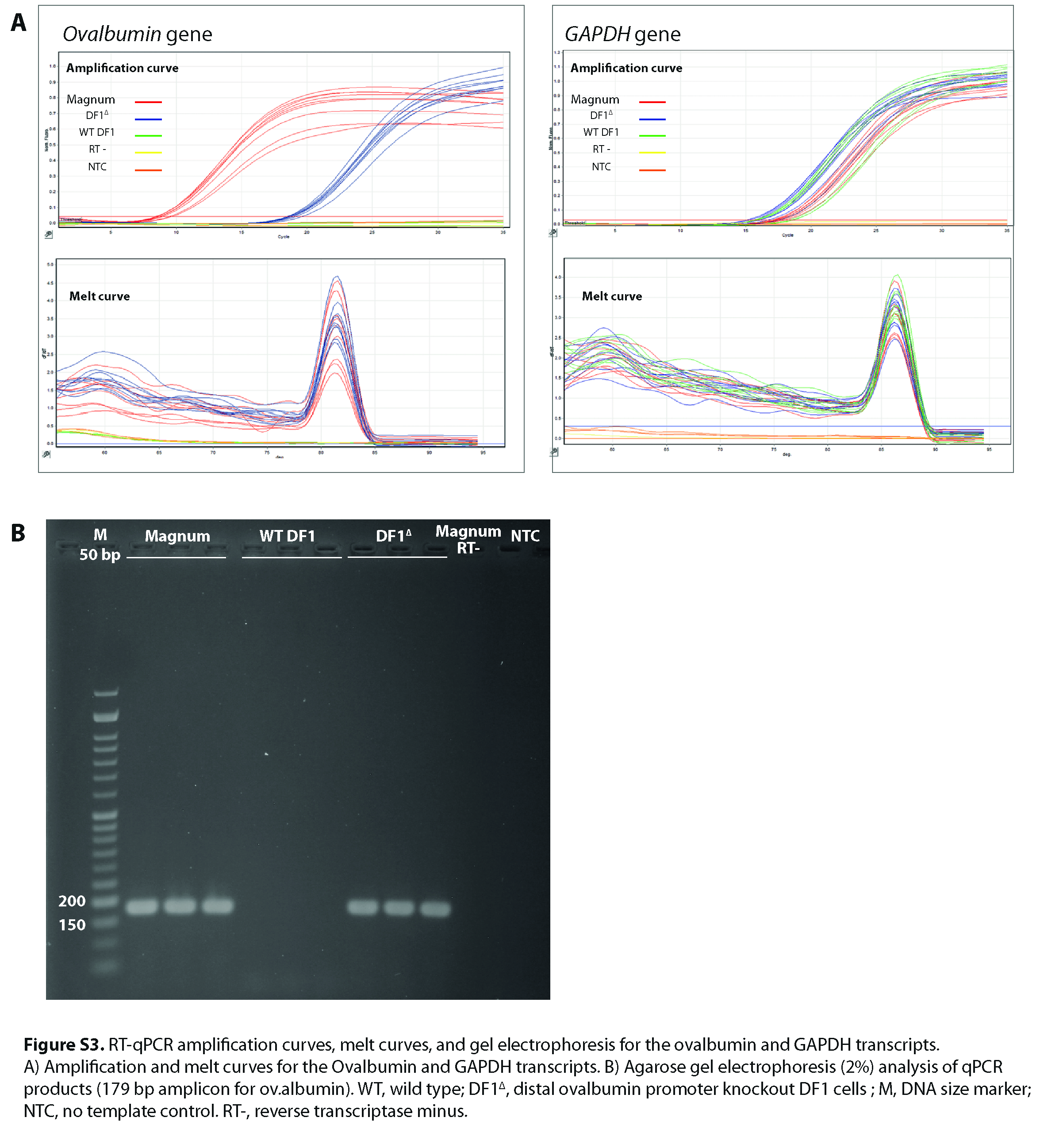

Supplement: Supplementary file 3 — Additional file 3. [file 13036_2023_367_MOESM3_ESM.tif]

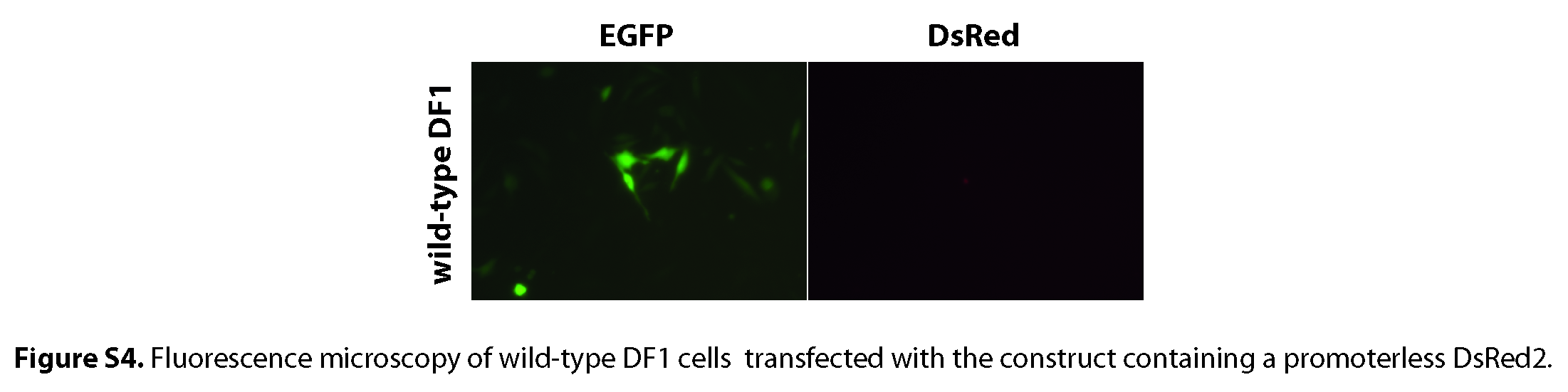

Supplement: Supplementary file 4 — Additional file 4. [file 13036_2023_367_MOESM4_ESM.tif]

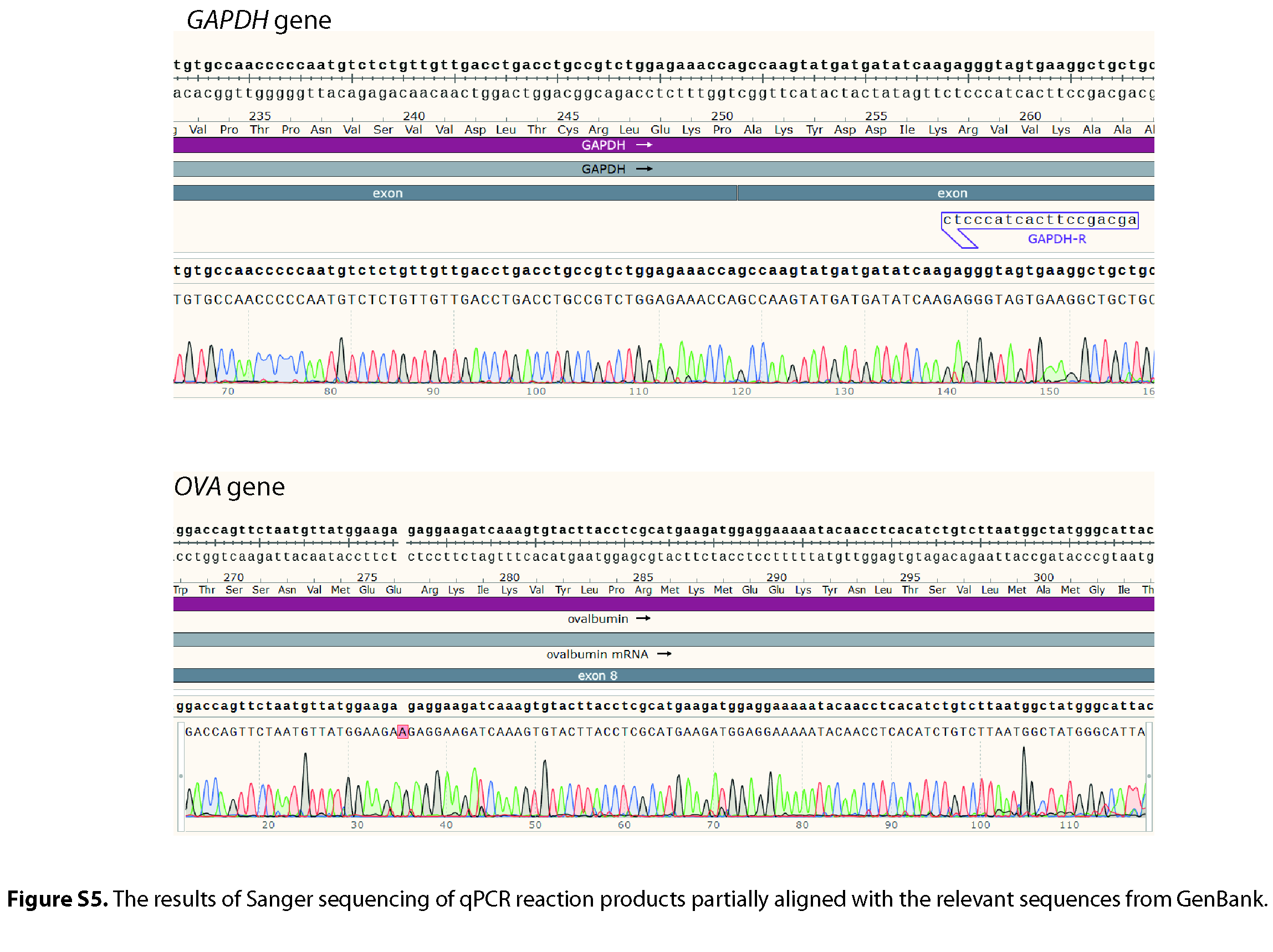

Supplement: Supplementary file 5 — Additional file 5. [file 13036_2023_367_MOESM5_ESM.tif]

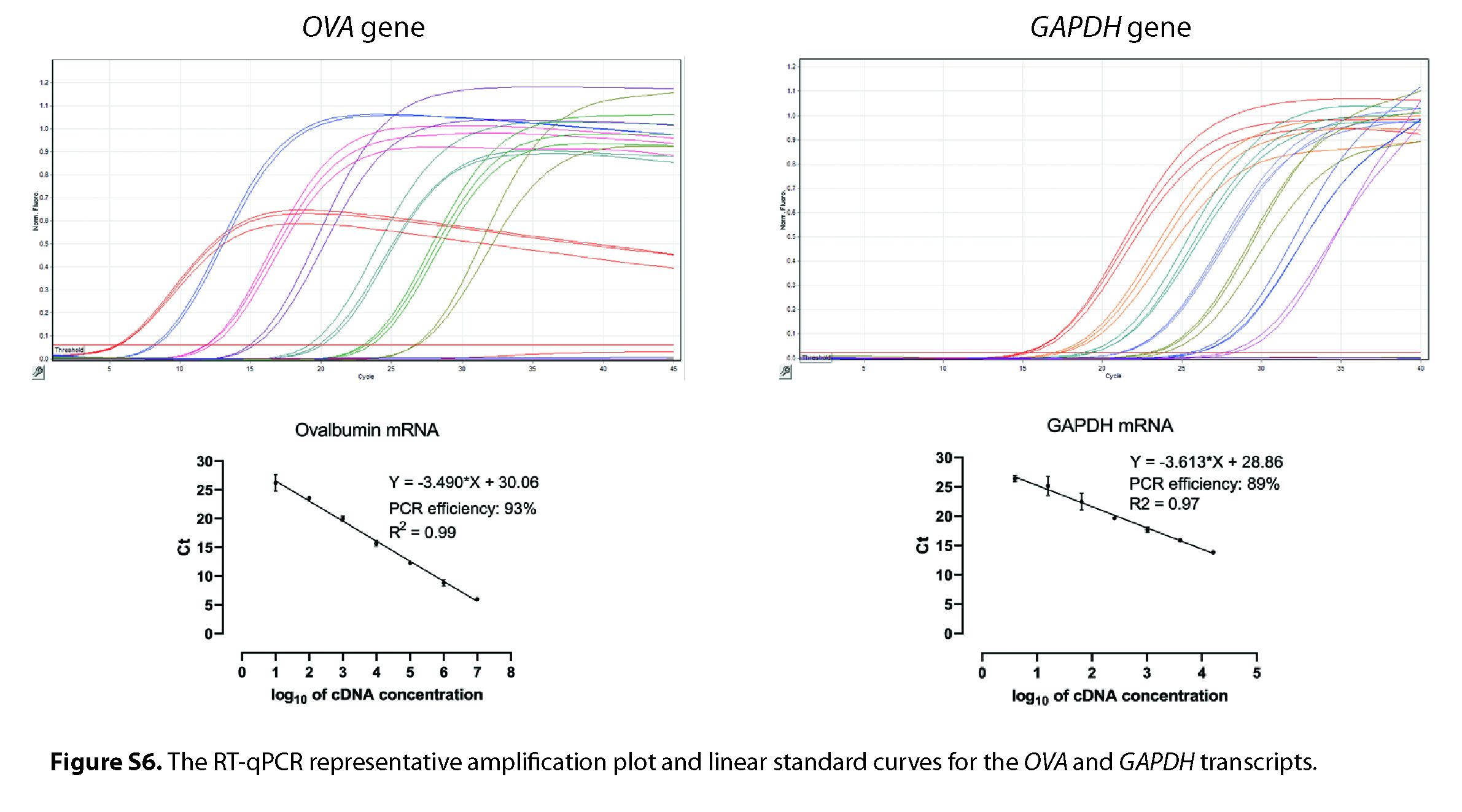

Supplement: Supplementary file 6 — Additional file 6. [file 13036_2023_367_MOESM6_ESM.tif]

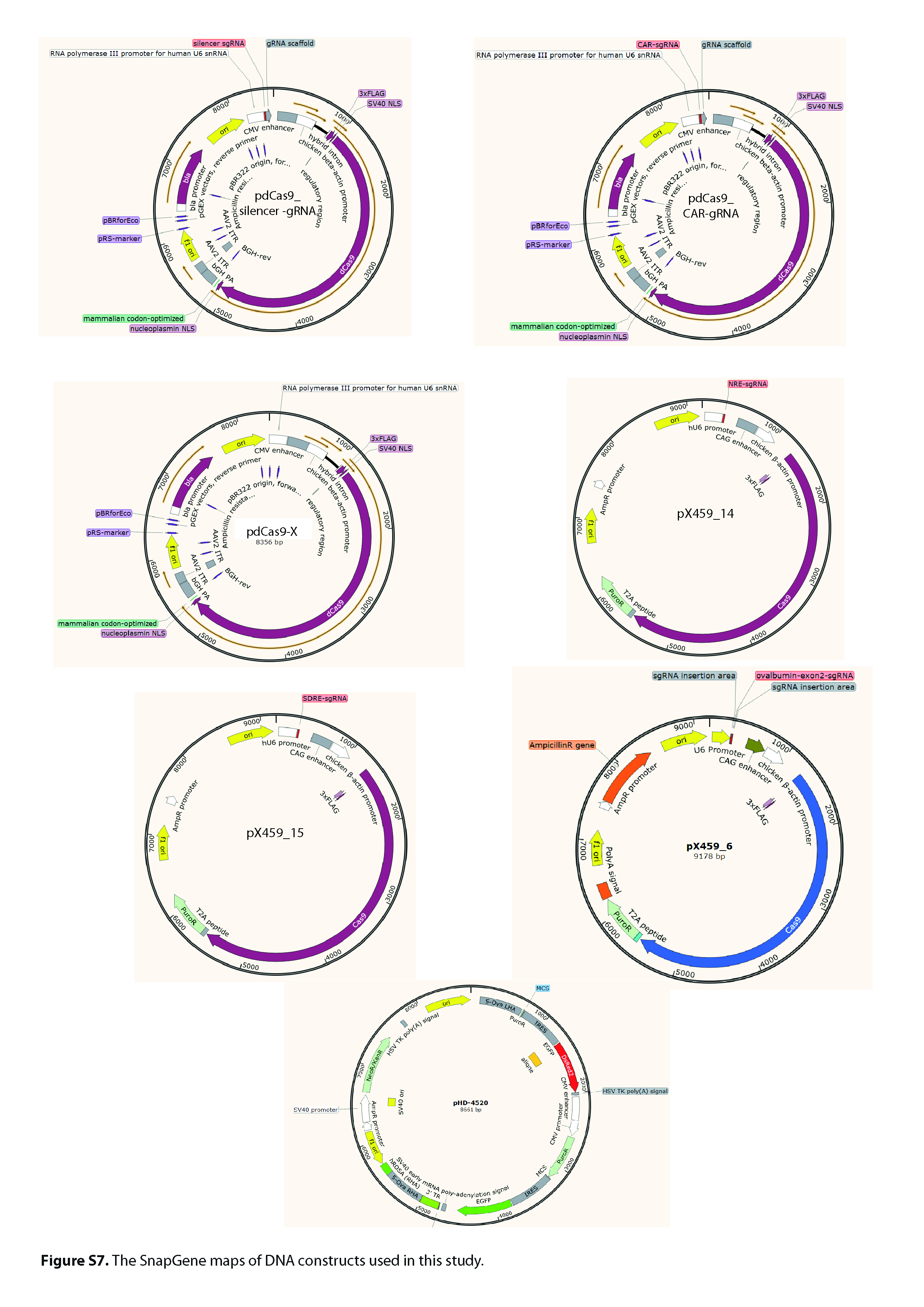

Supplement: Supplementary file 7 — Additional file 7. [file 13036_2023_367_MOESM7_ESM.tif]
